# Pre-analytical and analytical variables that influence urinary volatile organic compound measurements

**DOI:** 10.1371/journal.pone.0236591

**Published:** 2020-07-31

**Authors:** Michael McFarlanE, Ella MozdiaK, Emma Daulton, Ramesh Arasaradnam, James Covington, Chuka Nwokolo

**Affiliations:** 1 Department of Gastroenterology, University Hospitals Coventry and Warwickshire, Coventry, United Kingdom; 2 School of Engineering, University of Warwick, Coventry, United Kingdom; 3 Department of Health Sciences, University of Leicester, United Kingdom; 4 Faculty of Health Science, University of Coventry, United Kingdom; NIH, UNITED STATES

## Abstract

There has been rapidly accelerating interest in the utilization of volatile organic compounds (VOCs) as non-invasive methods for rapid point-of-care medical diagnostics. There is widespread variation in analytical methods and protocols, with little understanding of the effects of sample storage on VOC profiles. This study aimed to determine the effects on VOC profiles of different storage times, at room temperature, prior to freezing, of sealed urine samples from healthy individuals. Analysis using Field Asymmetric Ion Motility Spectrometry (FAIMS) determined the alterations in VOC and total ion count profiles as a result of increasing room temperature storage times. Results indicated that increasing exposure time to room temperature prior to freezing had a threefold effect. Firstly, increased urinary VOC profile variability, with a plateau phase between 12 and 48 hours, before further degradation. Secondly, an increase in total ion count with time exposed to room temperature. Finally, a deterioration in VOCs with each sample run during the analysis process. This provides new insight into the effect of storage of urine samples for VOC analysis using FAIMS technology. Results of this study provide a recommendation for a 12-hour maximum duration at room temperature prior to storage.

## 1. Introduction

The last decade has seen a rapid expanse of research into the utilisation of gaseous phase volatile organic compounds (VOCs) as biomarkers of disease across a range of medical domains [1]. Interest in this field has risen due to several advantages, including its low test cost, ease of use and mobility, potentially allowing point of care disease detection [2–6]. This has led to the VOC analysis of almost all types of biological sample that can be extracted from the body, with breath, urine, faeces, sweat and blood being the most common mediums. These media have been tested as a potential source of VOCs to diagnose a wide range of diseases, including gastrointestinal (Crohn’s Disease, Ulcerative Colitis, Coeliac disease), metabolic conditions (Diabetes Mellitus) and cancer [1, 2, 5, 6].

Our group has extensively investigated the use of VOCs across a wide range of gastrointestinal and metabolic disorders, principally from urine samples [7–20]. It is our opinion that urine represents the most acceptable and user-friendly medium, for both patients and the investigating team. A standard operating procedure for urine handling for analysis has been established by our research group and has been described previously [21]. If urine VOC diagnostics is going to transcend the clinical environment, then sample collection and storage must balance VOC stability with practicality for the patient and healthcare setting.

There is a paucity of published evidence to guide best practice in utilisation and storage of urine samples. Previous studies around storage have focused primarily on the liquid phase, utilising, for example, Mass Spectrometry (MS) and nuclear magnetic resonance (NMR) technology [22–24]. Only one study investigated the effects of long-term storage of urine samples, in this case using FAIMS (field asymmetric ion mobility spectrometry) analysis. Sagar et al found VOC signal degrade after 9 months of storage at -80 degrees [2]. Several studies have evaluated the effect of 24 hours of aging on human and mouse urine samples using Gas Chromatography-MS (GC-MS) [25, 26]. They found both increased and decreased urinary VOC concentration with age, attributing these findings to evaporation over time depending on the sample water content [26].

In order to increase our knowledge of VOC derived diagnostics, there needs to be a greater understanding of the role of sample storage on test sensitivity and specificity. Currently there are few evidence-based recommendations in this subject area. The aim of this study was to investigate the relationship between time at room temperature prior to analysis and observed urinary VOC concentration and variability using a FAIMS analytical method.

## 2. Methods

Scientific and ethical approval was granted by the University Hospitals Coventry and Warwickshire (UHCW) Research and Development Office, as well as the Warwickshire Ethics committee, ref: 09/H1211/38. Written informed consent was obtained from all study participants.

### 2.1. Patients

27 healthy volunteers were recruited from UHCW, a full medical and dietary history was taken. Those with gastrointestinal (GI) or urinary tract pathology and/or recent antibiotic use were excluded.

### 2.2. Urine collection, storage and transfer

Urine samples were collected from patients directly. They were immediately split into 6 aliquots and sealed into universal storage bottles. Sealing the samples minimised evaporation, a factor used to explain the altered VOC profiles in previous studies [25, 26]. Aliquots were then stored at room temperature for increasing periods of time (0, 12, 24, 36, 48 and 72 hours) prior to freezing at -80°C. Samples analysis occurred at the School of Engineering, University of Warwick, UK.

### 2.3. FAIMS analysis

In this study a commercial FAIMS instrument was utilized (Lonestar, Owlstone, UK). The mechanism of FAIMS has been described by our group previously [21]. This instrument was used over more traditional analytical approaches as samples can be tested quickly, at low test cost and it has shown promise as a VOC analyser for disease diagnostics. The measurement principle is based on measuring the mobility of ionised molecules in high electric fields. VOCs are separated based on their mobility. Molecules with similar mobilities will overlap, but the combination/amount will be related to the output voltage. Simply put, the higher the voltage output at a specific dispersion and compensation voltage, the more abundances of molecules with that mobility. The Lonestar is operated at positive pressure, where a chemical of interest is pushed into the system, it is then ionized using a radioactive source (in this case Nickel-63). Following this, the ions are flowed between two plates where an asynchronous electric field is applied, comprising of a short/high pulse and a longer/low pulse, but where the length of pulse multiplied by the electric field strength is the same. This either attracts, repels or has no effect on the ions. Any ions that touch a plate lose their charge and are not detected. To balance this movement, a compensation voltage is added. A range of electric field strengths and compensation voltages are scanned through to create a 3D image of the sample. Both positive and negative ions are measured resulting in 55,254 data points per sample. Our instrument has been connected to a commercial auto-sampler (Gerstel MPS, Germany). This allows automation of the sample testing, where each sample is heated to 40°C and shaken for 10 minutes before being tested dynamically, with the flow across the sample being set to 500ml/min, with a further 1500ml/min of make-up added before being sent to the instrument. In use 5ml of urine was aliquoted into a 10 ml vial and a crimp-top lid, with septum, added. The auto-sampler was filled with 14 samples with an air blank (empty vial) in between to reduce carry over between samples. Each sample was tested for a period of 120 seconds, which is equivalent to 14 number of full scans (where the compensation and electric field is cycled through). Once completed, the sample matrices were extracted from the blank controls.

**Fig 1:**
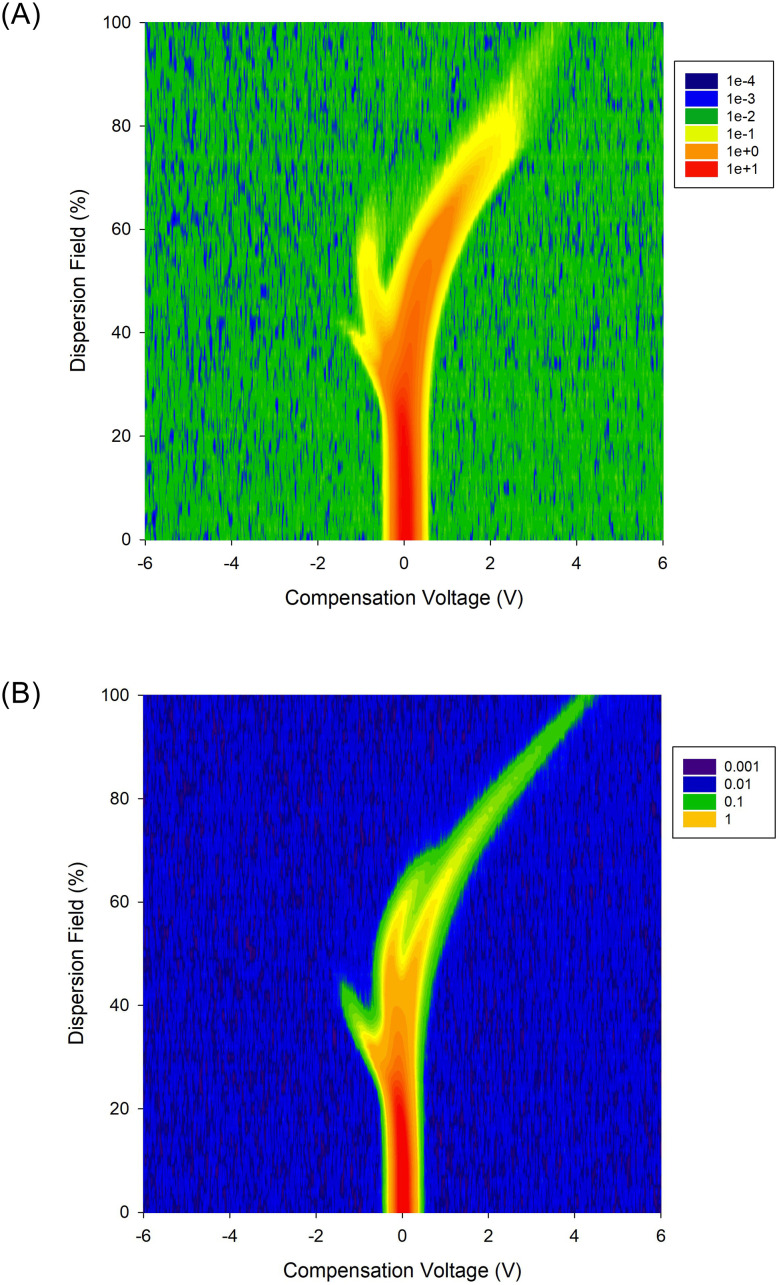
Examples FAIMS plumes for (a) positive ions and (b) negative ions, for the sample subject taken at the beginning of the study (note: both are showing absolute values). The plume contains chemical information, whilst the remainder is background.

Fig 1a and 1b give examples of positive and negative FAIMS plumes, which correspond to an individual matrix.

### 2.4. Statistical analysis

#### 2.4.1. Variation of mean of matrices from baseline

To analyse the urinary VOC variation from each subject, at each time point, the arithmetic mean of all positive and all negative matrices from time point zero, was compared to the arithmetic mean of the corresponding matrices for that patient at each time point, denoted by number of hours at room temperature (~20 to 22°C). A Spearman's rank correlation coefficient was utilised to test for a monotonic relationship between time and variation of mean matrices from baseline. This was done using the formula:

Variation(t)=∑pVp,t

Where:

$$\text{Vp,t}=\frac{\sum \text{c}\sum \text{s}|\sum \text{nMp,t,n,c,s}-\sum \text{nMp},\text{t}=0,\text{nc},\text{s}|}{\sum \text{c}\sum \text{s}|\sum \text{nMp,t}=\text{o},\text{n},\text{c},\text{s}|}$$

Notation used: Matrix—Mp,t,n,c,s; p—Patient number; t—time (0,12,24,36,48 or 72 hours); n—spectrum number at each time point (1–14); c—compensation voltage index; s—Separation field index; P—total number of patients: 20; T—number of time points: 6; N—number of spectra for each sample: 14; V–variation; C—total ion count.

The matrix represents the VOC signature of that sample at that time point, the purpose of this analysis was to see if the VOC signature significantly changed with increasing time at room temperature prior to analysis.

#### 2.4.2. Variation of total ion count from baseline

The relative variation in the total ion count in the FAIMS matrices across time was also calculated. The arithmetic mean of the number of ions detected for each patient, at each time point, was calculated. These means were then compared to the mean ion count, at time zero, for that patient. Spearman's rank correlation coefficient was used to test for a relationship between time and the mean total ion count variation from baseline.

Given by:

$$C_{t}=\;\frac{\sum_{p}C_{p,t}}{P}$$

Where:

$$C_{p,t}=\;\frac{\sum_{n}\sum_{c}\sum_{s}\left|M_{p,t,n,c,s}\right|}{M_{p,t=0,n,c,s}}$$

This analysis assessed whether any observed change in VOC signature reflected a net loss or gain of total ions in the sample or whether it was more due to a redistribution of ions.

## 3. Results

27 healthy subjects were recruited to provide urine samples. 1 did not return samples, 2 subjects provided insufficient urine for six aliquots and 4 samples were incompletely analysed due to technical errors with the Lonestar instrument at the end of the measurement process. A per protocol analysis was performed on the cohort of 20 complete sample sets.

The mean age of subjects was 49.6 years (Standard Deviation (SD) 9.6 years), and there were 6 males (23%). The mean BMI was 28 (SD 5.8) and there were 2 smokers (7.7%). Average alcohol consumption was 5.3 units per week per subject (SD 5.1).

### 3.1. VOC variability with time

**Fig 2:**
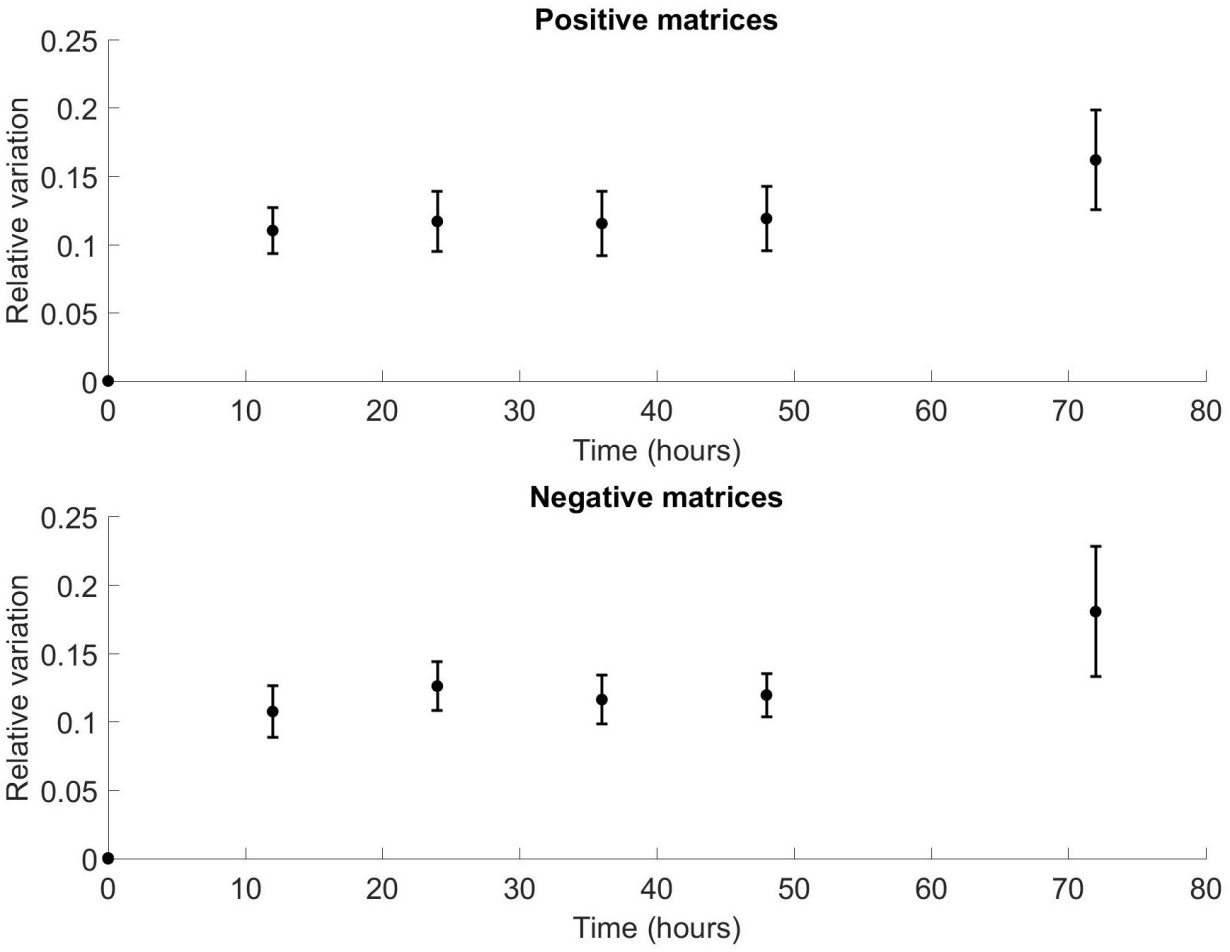
(a) Variation of positive FAIMS matrices from the mean of the matrices from t = 0. Bars show standard error. Correlation of mean variation with time: Spearman ρ0.94, p = 0.017. Correlation of all points with time ρ0.52, p < 0.001. Plateau phase seen between 12 and 48 hours. (b) Variation of negative FAIMS matrices from the mean of the matrices from t = 0. Bars show standard error. Correlation of mean variation with time: Spearman ρ0.83, p = 0.058. Correlation of all points with time ρ0.54, p < 0.001. Plateau phase seen between 12 and 48 hours.

**Fig 3:**
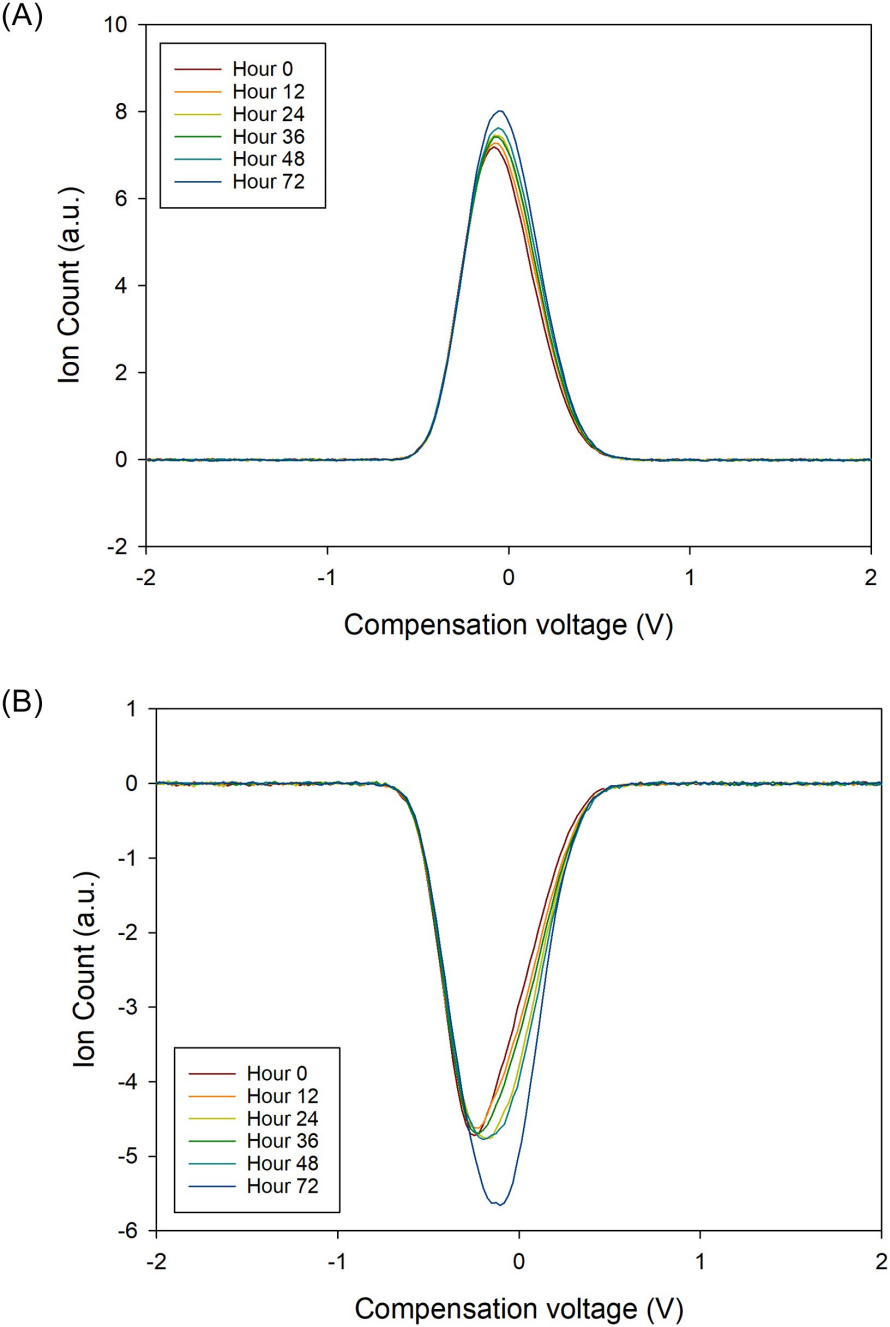
2D plot of compensation voltage versus on count at a single dispersion field and over time for (a) positive ions and (b) negative ions.

**Fig 4:**
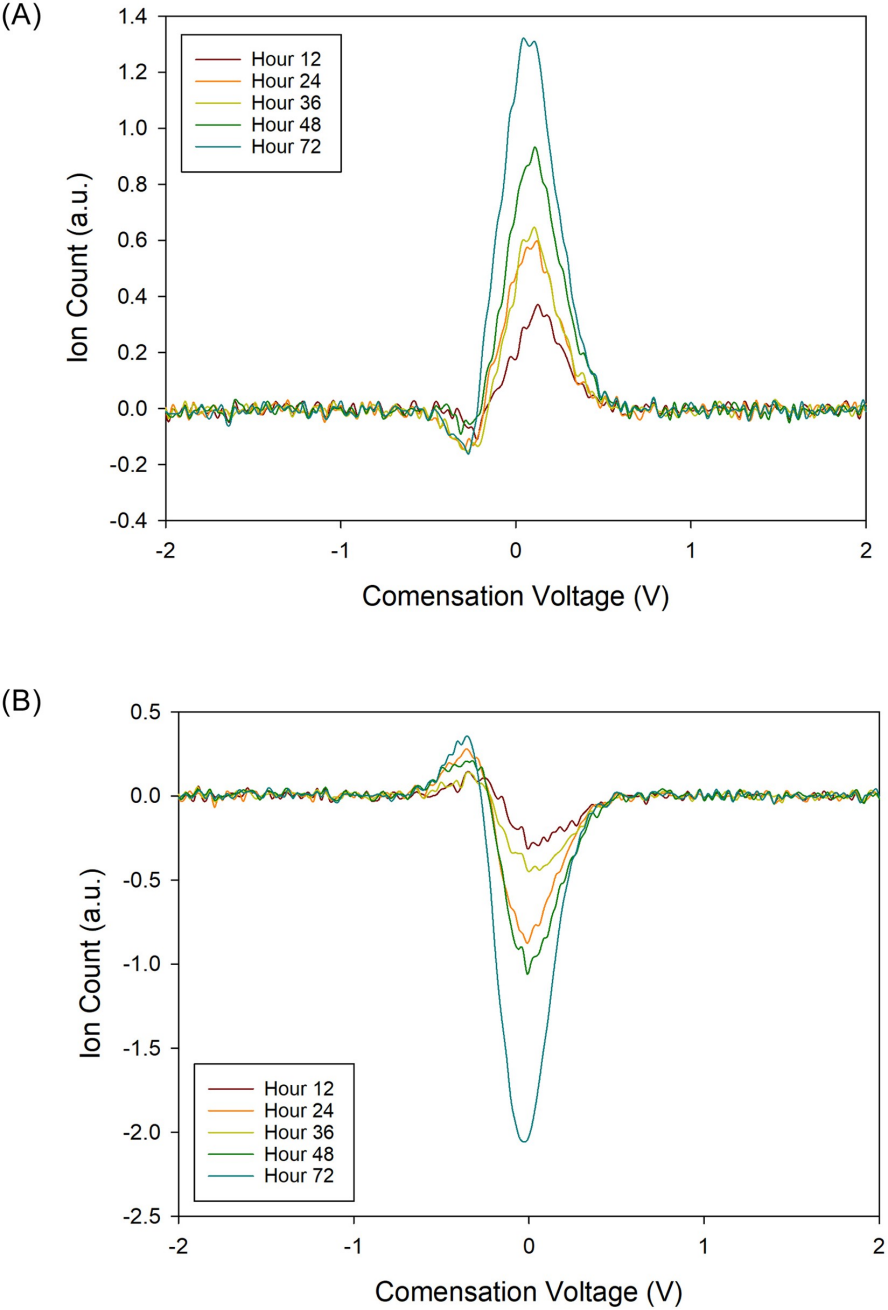
2D plot of compensation voltage versus on count at a single dispersion field and over time for (a) positive ions and (b) negative ions, showing different from hour 0.

When comparing matrix variability between samples, the relative VOC signature variation increased from time zero to 72 hours, for both positive and negative matrices. There was a plateau phase between 12 and 48 hours. This pattern was observed across all subjects, for positive and negative matrices. Fig 2a and 2b show the relative variation in the mean of the positive and negative FAIMS matrices respectively, compared to time. To emphasise this, Fig 3 shows a 2D plot at a single dispersion field (24%), for the same patient at all time points. At this dispersion field, there is not sufficient field strength to cause ion separation and therefore we get a plot of all the VOCs. In Fig 4, we have subtracted the time zero value from all the other hour points to make the differences more observable.

Spearman's rank correlation coefficient analysis gave a value of ρ0.94 (p = 0.017) for positive matrices, and ρ0.83, (p = 0.058) for negative matrices. When matrix data was pooled, Spearman's rank correlation gave ρ0.52 (p < 0.001) for positive matrices and ρ0.54 (p < 0.001) for negative matrices.

**Fig 5:**
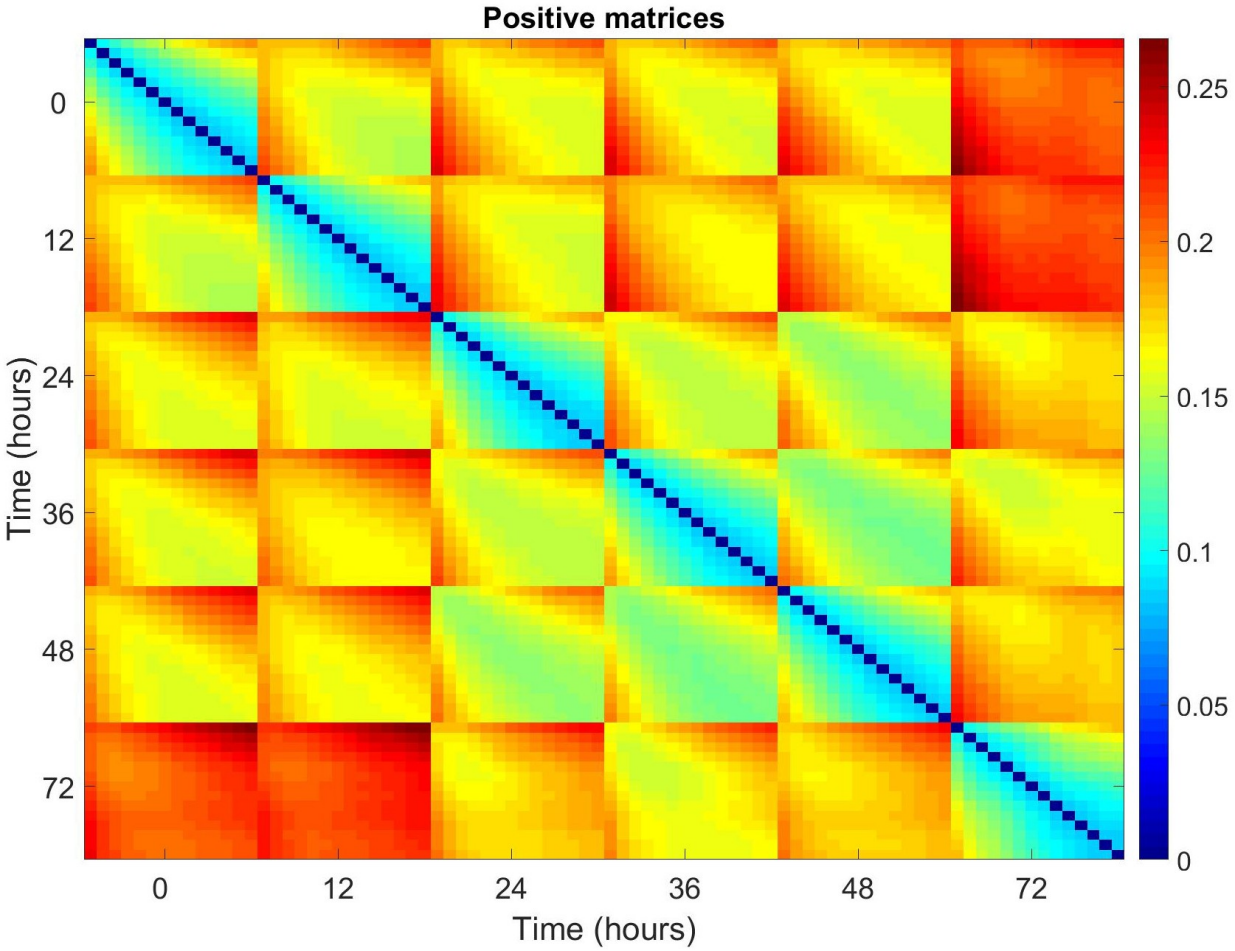
Pairwise relative differences for all pairs of positive matrices averaged across all patients. Blocks correspond to labelled times, with 14x14 elements corresponding to the associated matrices. Each 14x14 block corresponds to the pairwise differences between the matrices at each time point i.e. Time point 0, matrix 1 vs time point 0, matrix 1/2/3/4 etc.

**Fig 6:**
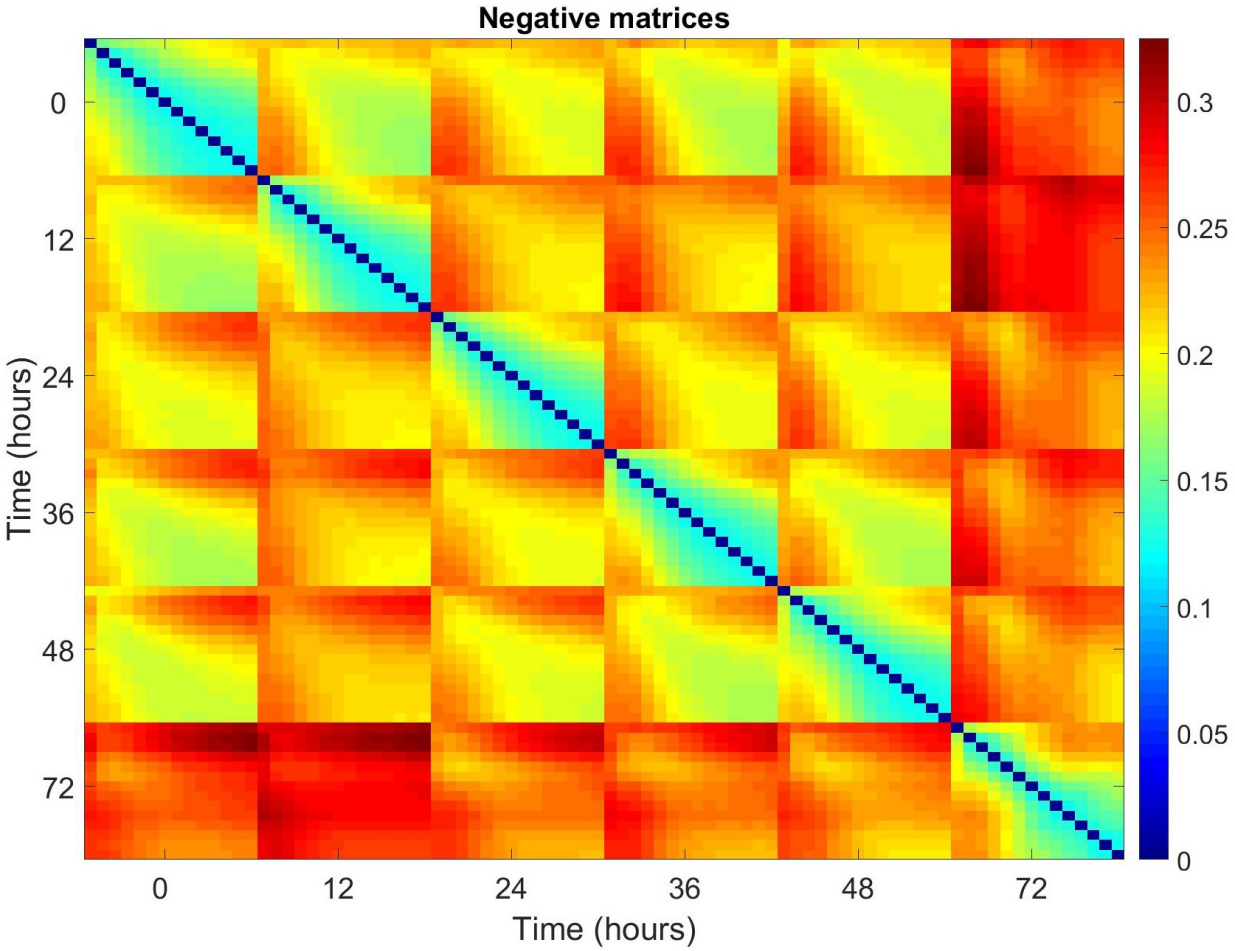
Pairwise relative differences for all pairs of negative matrices averaged across all patients. Blocks correspond to the labelled times, with 14 x 14 elements corresponding to the associated matrices.

To try and identify the source of the observed variation over time, average relative pairwise differences for all patients, across the 14 matrices, at each time point, were plotted for positive and negative matrices (Figs 5 and 6). Each 14x14 block corresponds to the pairwise differences between the matrices at each time point i.e. Time point 0, matrix 1 vs time point 0, matrix 1/2/3/4 etc. The equation for this is given below.

$$D_{t,n}=\left|V_{t1,n1}-\;V_{t2,n2}\right|$$

Where *V*_*t1*,*n1*_ is the variance at one time point and one matrix and *V*_*t2*,*n2*_ is the variance at the second time point and matrix (with time from 0 to 72 hours and matrix 1 to 14). The variance is calculated using.

$$V_{t1,n1}=\;\frac{\sum_{p}V_{p,t1,n1}}{P}$$

$$V_{p.t1.n1}=\;\frac{\sum_{c}\sum_{s}\left|M_{p,t1,n1,c,s}-\;M_{p,t=0,n=1,c,s}\right|}{M_{p,t=0,n=1,c,s}}$$

The structure of the block indicates that there is significant variation within the 14 matrices of any given time point. The difference between the first matrix at a time point and the subsequent matrices from the same time point grows with matrix number. This variation from baseline also grows with increasing time point from time point 0.

To investigate this further, the relative variation in matrices between the 14 matrices from each time point was calculated for both positive and negative matrices.

Mathematically this was done by:

Variation

$$\text{t,n}=\frac{\Sigma \text{p}\;\text{Vp},\text{t},\text{n}}{\text{P}}$$

Where:

$$\text{Vp,t,n}=\frac{\sum \text{c}\sum \text{s}|\text{Mp},\text{t},\text{n},\text{c},\text{s}-\sum \text{nMp},\text{t},\text{n}=1,\text{c},\text{s}|}{\sum \text{c}\sum \text{s}|\text{Mp},\text{t},\text{n}=1,\text{c},\text{s}|}$$

**Fig 7:**
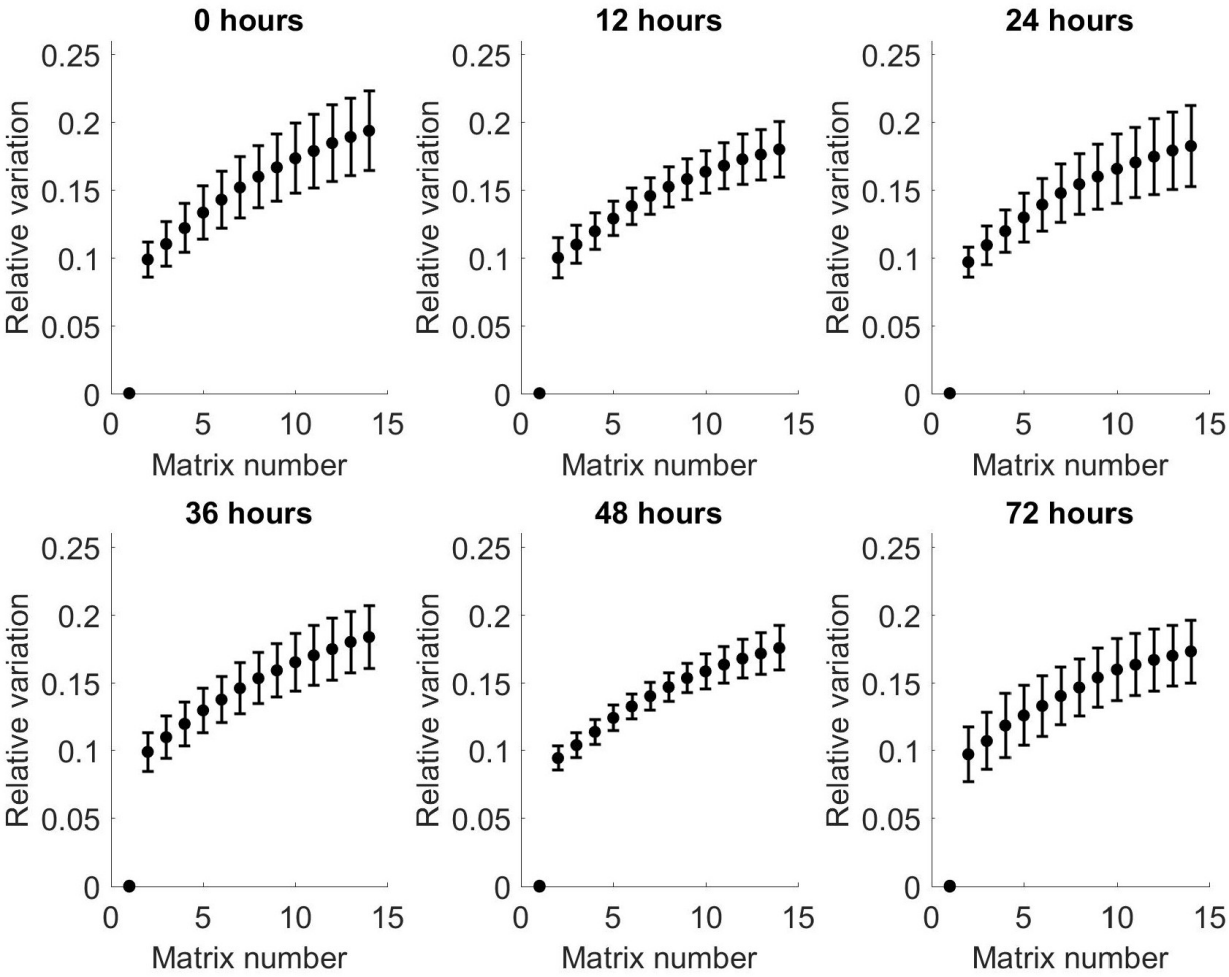
Relative variation from baseline of arithmetic mean of all patients as a function of matrix number for each time point for positive matrices. Bars show standard deviation.

**Fig 8:**
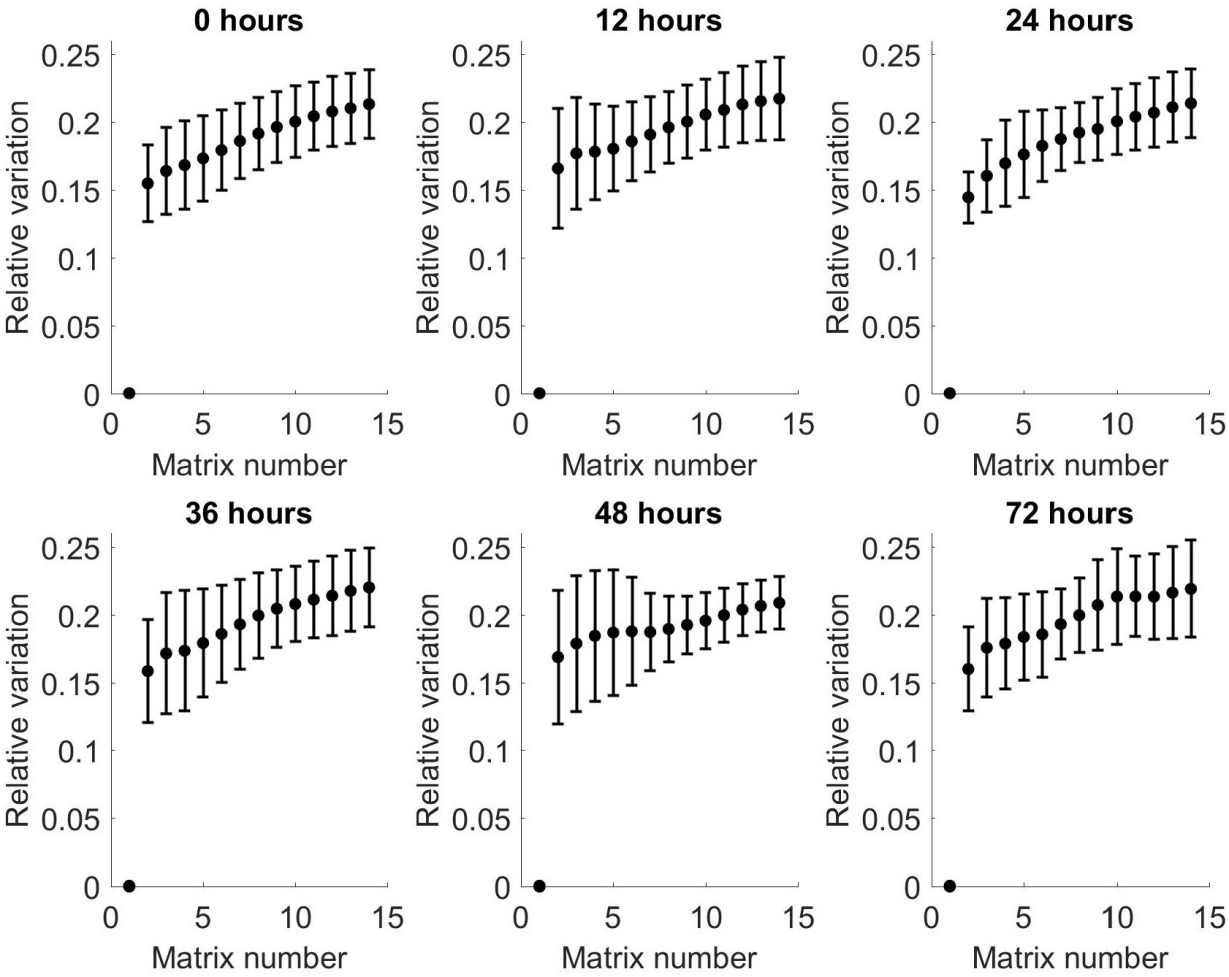
Relative variation from baseline of arithmetic mean of all patients as a function of matrix number for each time point for negative matrices. Bars show standard deviation.

Figs 7 and 8 show the relative variation in averaged matrices of all patients for positive and negative matrices, respectively, compared to matrix number. These show an increase in relative variation for both positive and negative matrices across all time points, with increasing matrix number. Comparison of the individual matrices at each time point showed there is a predominantly negative difference in spectra between matrix 1 and 14, indicating a loss of VOCs with increasing matrix number within each time point. These findings are replicated in the negative matrices.

### 3.2. Relative variation in total ion count

We also calculated the arithmetic mean of the sum of the number of ions detected for each patient at each time point and compared that to the average of the sum of the number of ions detected for that patient at time zero (*C*_*t*_). Given by:

$$C_{t}=\;\frac{\sum_{p}C_{p,t}}{P}$$

Where:

$$C_{p,t}=\;\frac{\sum_{n}\sum_{c}\sum_{s}\left|M_{p,t,n,c,s}\right|}{M_{p,t=0,n,c,s}}$$

**Fig 9:**
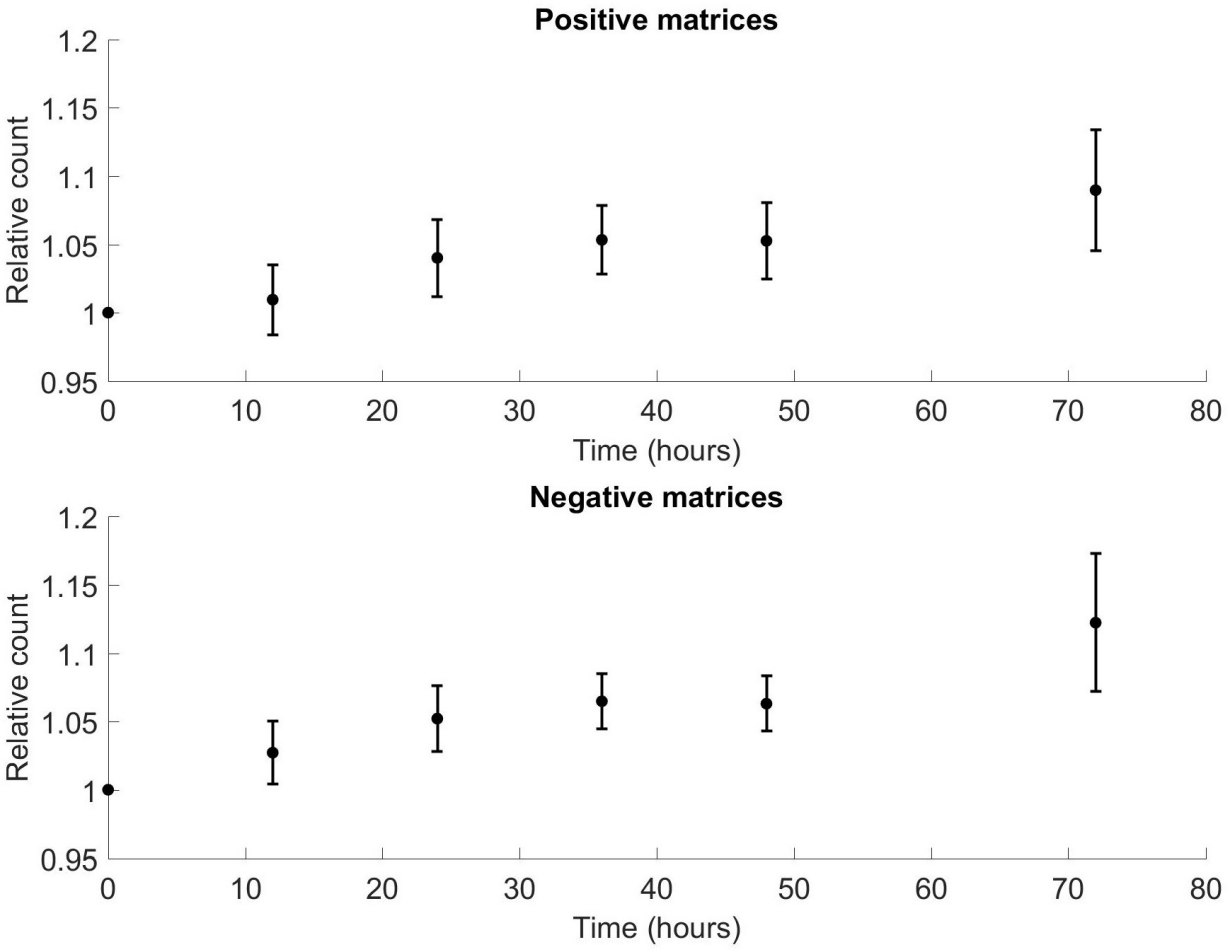
(a) Variation in relative ion count for positive matrices from mean of matrices at t = 0. Bars show standard error. Correlation of mean ion count with time: Spearman ρ0.94, p = 0.017; correlation of all points with time: Spearman ρ0.25, p = 0.009. Plateau phase seen between 12 and 48 hours. (b) Variation in relative ion count for negative matrices from mean of matrices at t = 0. Bars show standard error. Correlation of mean ion count with time: Spearman ρ0.94, p = 0.017; correlation of all points with time: Spearman ρ0.27, p = 0.004. Plateau phase seen between 12 and 48 hours.

The total ion count was found to increase over time for positive and negative matrices. This is shown in Fig 9a and 9b for positive and negative matrices respectively. Spearman's rank correlation coefficient analysis showed ρ0.94, p = 0.017 for positive matrices, and ρ0.94, p = 0.017 for negative matrices. When all patients were combined as a single time series, this gave ρ0.25, p = 0.009 for positive matrices, and ρ0.27, p = 0.004 for negative matrices.

**Fig 10:**
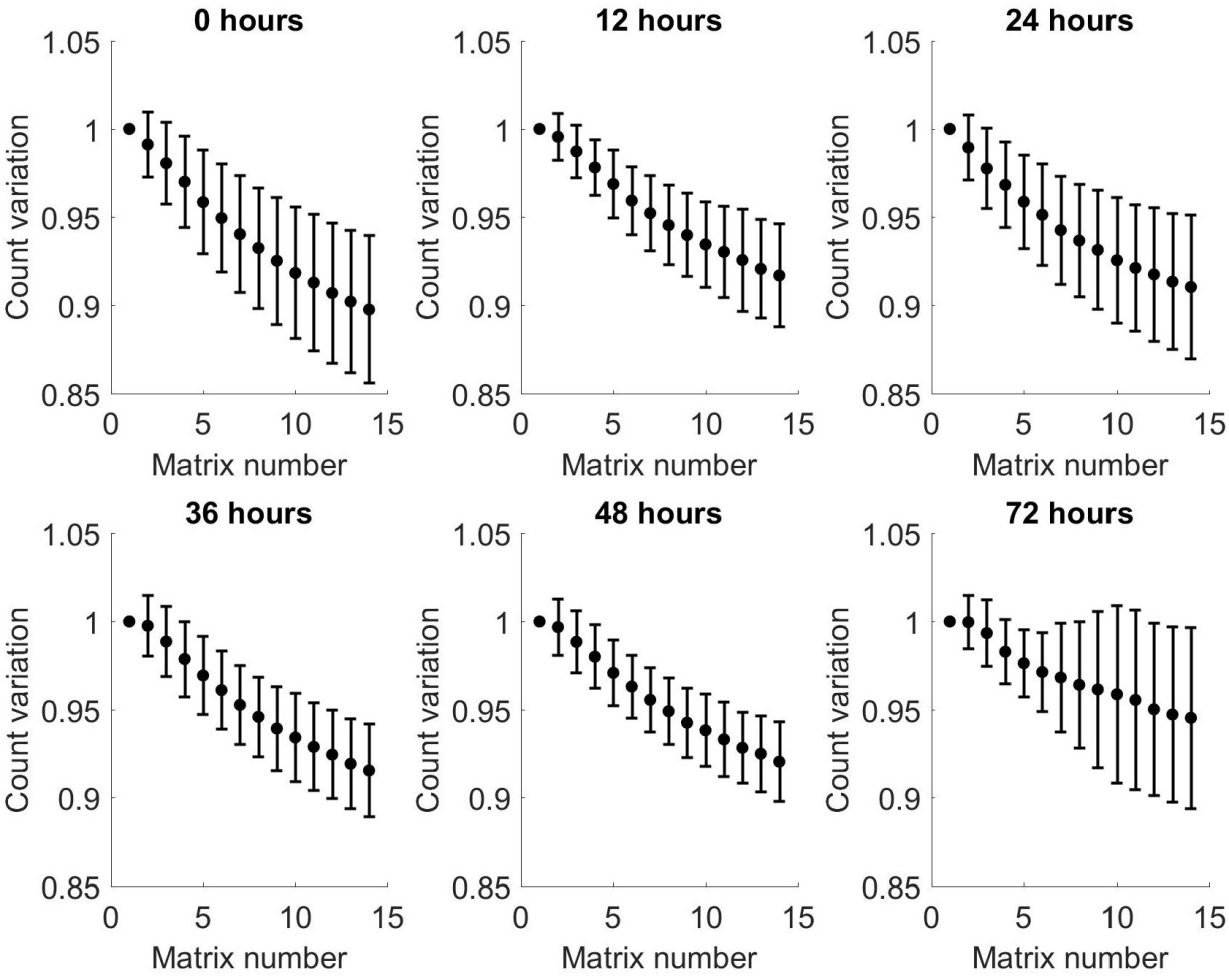
Relative variation of ion count from baseline of arithmetic mean of all patients as a function of matrix number for each time point for positive matrices. Bars show standard deviation.

**Fig 11:**
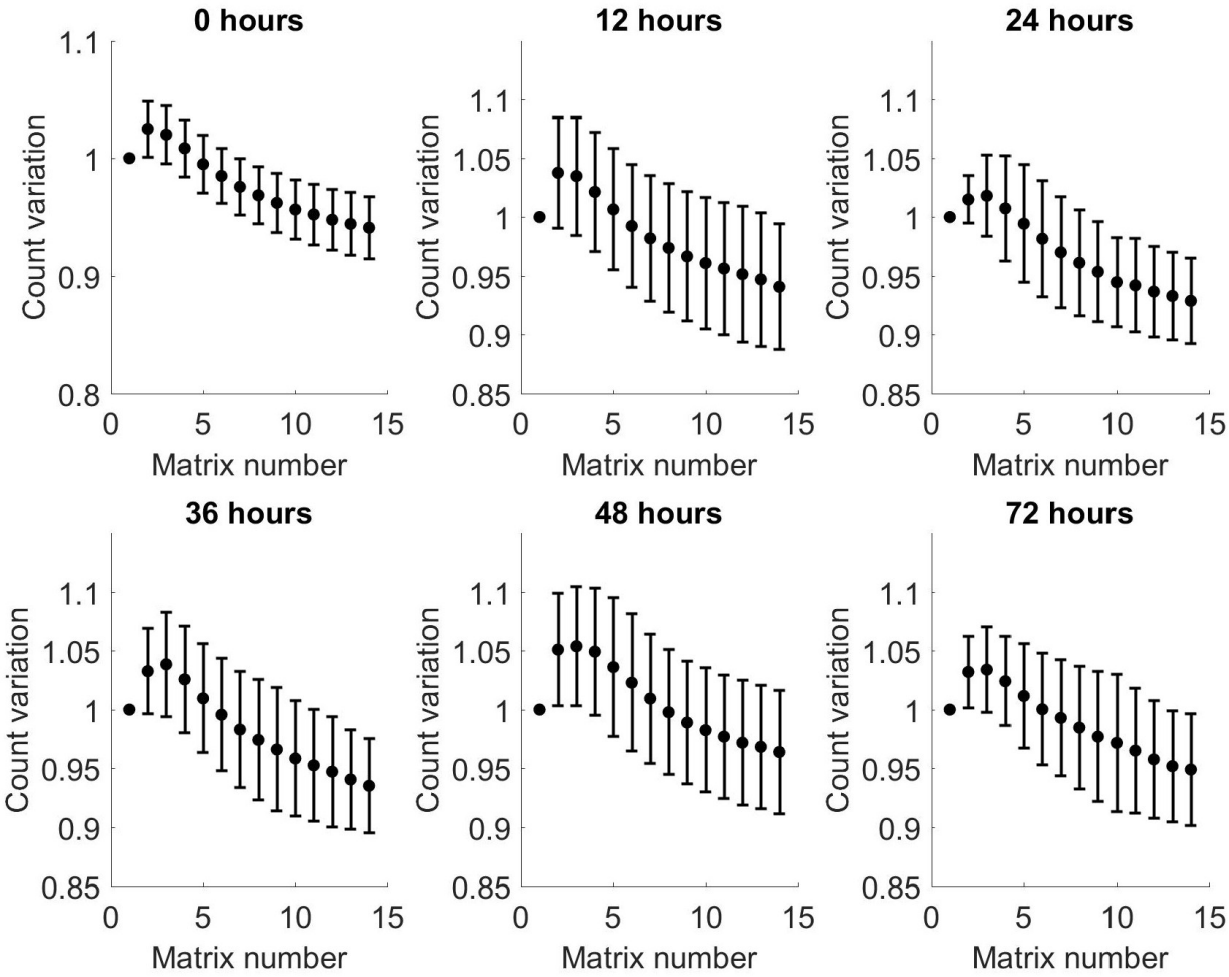
Relative variation of ion count from baseline of arithmetic mean of all patients as a function of matrix number for each time point for negative matrices. Bars show standard deviation.

Given that there is increasing variation when comparing sequential matrices for individual samples at a given time point described in the previous section, an analysis was run on the total ion count variation for sequential matrices from each time point. This showed that the relative ion count reduced monotonically for positive matrices, with increasing matrix number at the same time point, and increased transiently for negative matrices before also reducing. These results are shown in Figs 10 and 11.

## 4. Discussion

This study has demonstrated two clear patterns of change in urine samples analysed for VOCs, namely intra-individual variation over time and inter-individual variation with repeated analysis. This gives new insight into the net change in sample composition depending on pre-analytical factors, for example pre-freezing storage conditions and conditions during sample analysis.

This pattern was uniform across all subjects, suggesting that this is an intrinsic property of the VOCs. The presence of a plateau phase between 12 and 48 hours raises some questions. It may represent the VOCs, after initial degradation, reaching a "steady state", and only with exposure to room temperature beyond 48 hours does further degradation occurs. We are unable to determine whether this results from the sample being stored in a sealed container, and whether an open storage container would produce a different profile, although we speculate that it would be expected that an open container would have a far greater degree of degradation as it would promote evaporation and VOC loss, while a sealed container would inhibit both. The variation within the spectra increased with time. This suggests that the samples were degrading, reducing the overall number of ionised VOCs. The cause of this degradation over time is unclear. Urine was previously held to be a sterile substance, although this has recently been disproved [27]. All volunteers were healthy and given the consistent sample degradation pattern, it suggests that any bacterial contamination would have occurred across all samples. Therefore, whilst bacterial or environmental sample contamination from the urinary tract is plausible, it is improbable. There is a possibility it may derive from the universal bottles used for sample storage, although further studies using different storage vessels would be needed to further characterise this.

We also see a reduction in VOCs linked to repeated testing of the same sample. It suggests that VOC degradation continues during sample analysis. This leads to greater relative variation of the VOC profile from the first to last matrix of each time point, but an overall reduction in ion count number. This variation was consistent across all subjects and time points. The finding that total ion count reduces with increasing matrix number for each time point suggests that the ions and VOCs are depleted due to degradation over the course of serial analyses of the same sample. The most likely cause will be VOC depletion with repeated sampling, through a natural evaporation process. There is the possibility that this observation could be due to the prolonged room temperature exposure of the latter matrix numbers for each time point compared to the first. However, as the sample were held at 4°C before testing within the autosampler, this is unlikely.

Within the observed VOC signatures, it would be expected that individual VOCs would degrade at different rates which could differentially affect the VOC signature. The use of FAIMS as a measurement technique meant that identifying the nature of the VOCs that have broken down was not possible. Previous work has attempted to characterize and create a database of VOCs found in bodily secretions of apparently healthy individuals. They identified VOCs from several chemical families, including: hydrocarbons (e.g. alkanes and alkenes); primary and secondary alcohols; aldehydyes; ketones; esters; nitriles and aromatic compounds [28]. The authors compiled a list of 1840 VOCs from breath (872), saliva (359), blood (154), milk (256), skin secretions (532) urine (279), and faeces (381). They qualified that detection of chemicals in from some sources, such as skin, was very dependent on the medium used to collect the samples, in addition to the detection method used [28]. Further to this study, to potentially identify individual VOCs then studies using technology which can identify individual VOCs, such as MS technology, would be needed.

This study has far reaching practical implications for any further work performed using VOC analysis, as it affects sample collection and storage. Given the rapid degradation of VOC profile which occurs between 0 and 12 hours, and then the plateau which occurs between 12 and 48 hours, there are two potential strategies. The first is that only fresh voided samples are collected, and they must be frozen immediately to prevent degradation. This has logistical implications for sample collection in a research environment, and potentially less so in the clinical setting. The second option would be to allow subjects to produce their samples at their convenience, seal them immediately, and return the samples, provided this occurs within 12 hours of the urine being passed into the bottle. This would rely on a high level of patient education and compliance. This would also require that any freshly produced urine should be stored at room temperature, so that all samples are frozen at the same time. Based on the evidence presented in this study, the recommend sample storage at room temperature is no more than 12 hours prior to freezing in order to minimise inter-sample variability.

Future studies the initial 12 hour degradation period are needed, to determine when most of this signal alteration/degradation occurs. As an initial pilot study we selected the time points used, as these were the most realistic integers seen in real life sample collection. Samples are either frozen immediately on production, or over the course of the same or next day.

This study has not assessed potential confounding variables such as gender, age and diet which are known to contribute to VOC signatures and individual VOC content. The sample size of this pilot study did not allow for sub-group analysis as there would have been a high likelihood of Type 1 error and so we analysed the group as a whole.

These results are consistent with findings from previous studies looking into the effects of storage on VOC profiles [24, 25]. Although previous work was performed on non-sealed samples and the alterations were principally attributed to evaporation of water from the samples, which has been minimised here by using sealed storage containers.

This study was conducted on healthy subjects, and there is currently no available research into the degradation of VOCs in diseased subjects. It is not known if the degradation follows a similar pattern, as in healthy subjects and if diagnostic potential is lost. Furthermore, this study had a sample size of 20, hence larger scale studies over a longer time period and with several interim time points are needed. Future studies should also allow for full mapping of the degradation patterns of VOCs in urine, tracking specific chemicals with prolonged exposure to atmospheric temperature prior to freezing for subsequent analysis.

## 5. Conclusions

This study is the first to demonstrate an increase in variation of urinary VOCs and total ion count after increasing exposure to room temperature using FAIMS analysis. Given the findings here, we recommend that in future, urine intended for VOC analysis should be frozen within 12 hours of voiding to prevent excessive sample degradation and loss of signal. Further studies are needed into the initial 12 hour period to to determine when most of this signal alteration/degradation occurs. This study provides an important platform for further work into the effect of storage of urine samples for cases rather than controls, and recommendations have been made to improve handling of urine specimens for VOC-based disease detection. The overwhelming benefit of urine-based testing relates to patient satisfaction and hence high uptake. However, for this to transcend the clinical environment and make a real impact on diagnostics in medicine there needs to be a much greater understanding of the stability of this medium going forward.
